# Be Sanitary and Be Sane*Complimentary to the San Antonio Sanitary Association, and as a souvenir of the Belknap Rifles Lecture Benefit, Armory Hall, Tuesday Evening August 29, 1899.

**Published:** 1899-10

**Authors:** 


### "Be Sanitary and Be Sane."*

PRESENTED BY ALBERT E. HYDE, OF CHATTANOOGA, TENNESSEE, WRITER AND LECTURER ON THE SUBJECT OF "THE PREVENTION OF DISEASE."

To sustain its reputation as a health resort a city must protect its resident population from the diseases its climate is advertised to cure.

In a climate peculiarly antagonistic to certain disorders, residents

---

*Complimentary to the San Antonio Sanitary Association, and as a souvenir of the Belknap Rifles Lecture Benefit, Armory Hall, Tuesday Evening August 29, 1899.

of cities can only suffer through the carelessness or negligence of the municipal government.

What a sad commentary on the sanitary condition of San Antonio—the recognized Mecca for the afflicted of harsher climates—that its citizens are dying of diseases its location and climate are supposed to relieve.

That this is a fact no one can doubt who has read Dr. Paschal's statistical reports. He finds the death rate of San Antonio increasing in greater proportion than the growing population will excuse.

He is not to be condemned for having the courage and honesty to state facts in an effort to arouse the citizens to a sense of their personal danger as well as to protect, for commerical reasons, the fame of the city as a health resort.

On the contrary, he should be upheld and the city placdd in a condition of cleanliness and sanitation which would enable it to sustain its splendid reputation for all time.

The cause of the high death rate at San Antonio is the lack of proper municipal ordinances regulating filth and garbage disposal, street spitting and sweepings, disinfection and proper house sewage connections.

With less than fifteen hundred buildings, out of more than eight thousand in the sewer districts, connected, San Antonio can not long retain her reputation as a health resort abroad.

To fully insure public safety, a city can not afford to stop at sewer connections, filth and garbage disposal. To arrive at the maximum saving of human life, every foot and joint of every house drain and every fixture connected thereto must be known to be absolutely water and gas tight.

To determine this, houses already connected must be thoroughly inspected from the street sewer to the roof, and the balance of the houses connected as soon as possible under honest and efficient inspection. Before a shovel full of dirt is thrown upon a drain pipe, or they are concealed in walls or floors, they must be subjected, under ordinance, to the water test and after the fixtures are set, to the smoke test, before the safety of the family can be assured.

There is a law in this State which affords that protection to the citizens of every city. Why does San Antonio's municipal government fail to ratify that law? It takes the appointment of the inspector away from the municipal council, believing that it is not safe to trust the lives and health of the people to any political spoils system.

The Texas law creates a board of examiners to test the competency of the laborer who does the work and the inspector who inspects.

Remember that the most competent and fearless inspector can not make unskilled men do skilled work. He must in a measure accept conditions as he finds them. Unskilled labor must be removed from the field before an inspector can guarantee safety to the slumbering household.

We can not improve plumbing and drainage conditions until we improve the character of plumbing labor.

Such a law does not work a hardship upon any competent man. It does not restrict trade competition. It simply removes from the field incompetent and unskilled men, leaving the field open, however, to the keen competition of competent men. It will not encourage trust or combine. It will not place the industry of plumbing and drainage under the control of the men who successfully stand the examination. It is impossible to improve the present opportunity the plumber enjoys to defraud the people.

The strict enforcement of the Texas State law by every Texas city will remove the necessity for an association of plumbers, the object of which is to regulate prices.

It guarantees the people better health and greater economy; or, in other words, it will give them value received for every dollar invested in plumbing and drainage. No man is competent to say that the strict interpretation of this law will foster or encourage a combine or trust among plumbers, who has not given at least two or three years earnest study to the conditions out of which has grown the Master Plumbers' Association of the United States.

After almost nine years of careful study of the situation and an intimate association with the members of this organization, nearly three years of which were devoted to organizing plumbers on what is known as the "Southern League" plan, the writer deliberately declares that the Texas State law, ratified by the cities of Texas, and honestly enforced, will destroy the present opportunity of the plumber, through organization or otherwise, to take advantage of the public ignorance.

He further declares, that if he believed for an instant that such a law would assist the plumber in controlling prices, or discourage legitimate competition, as a citizen of Chattanooga and a native of the South, he would unhesitatingly denounce it from the house-top.

Until you establish the board of examiners authorized under the Texas State plumbing law, you cannot hope to reduce to any considerable extent the death rate of San Antonio.

San Antonio could afford to isssue bonds to the amount of two hundred and fifty thousand dollars, or even five hundred thousand if necessary, or levy a tax, to place the city in a condition of cleanliness and sanitation to protect resident population from diseases introduced by the visiting sick and to sustain the health reputation it now enjoys.

A prominent professional gentleman, and property owner, said to the mayor of the city: "If you will institute this sanitary system and issue bonds of a sufficient amount necessary to place this city under perfect sanitary regulations, I will make a personal canvass of the business men and demonstrate to them that the appropriation is not only justified as a wise measure in the interest of public health, but that it is also a commercial proposition that no business man in San Antonio can afford to oppose."

Here is the plan in detail:

### A MODEL CITY.

To reduce death rates and the expenses of sickness, the sanitary system of all cities should operate under a State law creating a board of plumbing examiners, not appointed by the mayor and board of aldermen.

The duty of this board of plumbing examiners is to pass upon the competency of those who apply for license to do plumbing and house drainage. To examine applicants for the office of chief inspector of plumbing and assistant inspectors.

A city should be provided as follows:

1. A system of sewers; separate system preferred.

2. A water supply not contaminated by the sewage of any city.

3. Sewage should be destroyed by either crematory process or used, after chemical or bacterial changes, for farm fertilizer.

4. A plumbing ordinance regulating plumbing and house drainage and house water supply.

5. A chief inspector of plumbing.

6. An ordinance requiring every householder to connect every house and hovel within the city limits to the sewer system.

7. An ordinance regulating filth and garbage disposal and the periodical cleaning and disinfection of back premises and alleys.

8. A sprinkling ordinance. No dust should be allowed in a model city. Dust carries the germ of consumption.

9. Street cleaning ordinance. The business streets should be thoroughly sprinkled and cleaned every night. Residence streets sprinkled and kept clean all the time.

10. A department of Public Health and Hygiene, having plumbing, drainage, disinfection, etc., under its supervision. Such a department should be equipped with every modern sanitary plumbing testing device and also the best apparatus for "formaldehyde gas disinfection." [For domestic uses the 40 per cent. solution of formaldehyde gas or "formalin" reduced in about the proportion of ten parts water to one part formalin is the best disinfectant known. It is a positive germicide and can be sprinkled freely on carpets, curtains, bedding or wall paper without injury. It should be used freely about sinks, bath rooms, lavatories, etc., or where there is a suspicion of filth or foulness.]

A plumbing work room, where applicants for plumbers' license and inspectorships could be practically as well as theoretically examined. This department should have the power to order house to house inspection. There should be scientific appliances for ascertaining the quality of air in houses. When unoccupied houses are found to be in dangerous condition they should be promptly condemned and owners notified to thoroughly repair plumbing and drainage system and thoroughly disinfect premises before property could be advertised for rent. This would keep families renting homes out of death traps. School houses should have janitor service during vacation, water being turned on at stated intervals to avoid the danger of trap evaporation or the fouling of water in them. Before the opening of schools the buildings should be thoroughly disinfected.

In such a city the poor who rent would have cleanly, healthful homes, properly connected to the public sewers and supplied with cheap but safe plumbing fixtures. The department of health would furnish rules and regulations for the proper care and attention which should be given such fixtures and drainage systems daily to save repairs and to prevent dangerous conditions of filth. Such rules subject to regular inspection by the chief plumbing inspector or his assistants.

A city so provided and cared for could not propagate acute infectious and contagious diseases, when dangerous conditions, the result of prior ignorance and neglect, were removed by house to house inspection. This city would be proof against the epidemics. From the very beginning of such reform, death rates and sickness would diminish rapidly.

What a wonderful thing is cleanliness! A man may be poor and unfortunate, but he can be clean! What an attractive place a clean home is! There is an atmosphere about it which breathes purity and peace! Clean individuals and clean homes make a clean city, and there is nothing so conducive to progress as a city which takes pride in its cleanliness! It means health for the people and health means everything that is desirable, for almost everything else lies within the power of its possessor. Clean sanitary cities in the South will attract the right sort of immigration and capital to take advantage of our incomparable inducements! The knowledge that we protect the lives of our citizens will do more to advertise us abroad than anything else!

Here is beautiful, historic, picturesque San Antonio, nature's health resort and flower garden, whose name is almost a household word in the nation! A climate of perpetual summer, kept fresh and invigorating by the salubrious salt breezes of the great gulf! The balmy air purified and rarified by the cooling breath of the northers!

Here are a wise people, condemning the open ditch and fetid cesspool and reclaiming their once beautiful river! A half million dollars have been expended in this praiseworthy enterprise, yet there seems to be a disposition on the part of your municipal government to defeat the purposes for which these underground canals were intended. What a shame to turn the awful air generated in them into your homes and public buildings!

Better have continued the winding river for uses for which it was never intended by the Creator. Better have retained the pestilence breeding cesspools, for then your salubrious climate had a chance to purify or nullify in a measure the bacteria-laden atmosphere!

Now, through defective house sewerage connections, these stealthy, odorless vapors will find direct access into your homes with no opportunity for salt breeze and sunshine disinfection!

Do not endanger the name of your fair city as a health resort through defective house drainage connections!

Start right with your sewers, and the fame of your fair city will spread over the whole world!

Pass a municipal quarantine against the contagion of the sewers under the Texas State law! Institute "house to house inspection" under municipal ordinance and you will build a foundation for commercial and industrial development without a flaw! Municipal sanitary ordinance, inspection and examination, under the State law, will prove the greatest and wisest investment this city has ever made!

Texas, to her honor and glory, was the first State south of the Ohio river to pass a sanitary law! Let San Antonio, her most illustrious city, be the first among her municipalities to conform to its every requirement!

